# Epinephrine levels decrease in responders after electroconvulsive therapy

**DOI:** 10.1007/s00702-021-02420-1

**Published:** 2021-09-23

**Authors:** Christoph Pollak, Hannah Benedictine Maier, Nicole Moschny, Kirsten Jahn, Stefan Bleich, Helge Frieling, Alexandra Neyazi

**Affiliations:** 1 Department of Psychiatry, Social Psychiatry and Psychotherapy, Hannover Medical School, Carl-Neuberg-Str. 1, 30625 Hannover, Germany; 2 Laboratory for Molecular Neuroscience, Department of Psychiatry, Social Psychiatry and Psychotherapy, Hannover Medical School, Hannover, Germany

**Keywords:** Catecholamine, Electroconvulsive therapy (ECT), Treatment-resistant depression, Major Depressive Disorder (MDD), Cortisol

## Abstract

We examined potential changes in catecholamine plasma levels and cortisol serum levels in 29 patients with treatment-resistant Major Depressive Disorder (MDD) during a course of electroconvulsive therapy (ECT). Blood samples were taken at three different time points: directly before (T1) and 15 min after (T2) the first ECT, and directly before the last one (T3). Patients responding to ECT had a significant decrease of the intraindividual epinephrine plasma level when both first time points (Δ T1-T2) were compared (*χ*^2^ (1) = 10.05, *p* = 0.002). This finding encourages further investigation in the understanding of the catecholamine-metabolism including its release and uptake in patients with treatment-resistant MDD receiving ECT.

**Supplementary Information:**

The online version contains supplementary material available at 10.1007/s00702-021-02420-1.

## Introduction

Electroconvulsive therapy (ECT) is considered an effective treatment option for patients with previous pharmacotherapy failure as well as for patients with Major Depressive Disorder (MDD) (Gaynes et al. 2008). ECT has been shown to affect the hypothalamic–pituitary–adrenal (HPA) axis by normalizing the results of the dexamethasone suppression test in a sub-group of severely depressed patients (Yuuki et al. 2005). The monoamine hypothesis suggests—among others—a deficiency of norepinephrine neurotransmission in the brain leading to depression (Ruhé et al. 2007). Studies have shown that suicidal subjects had significantly higher 24-h urinary levels of cortisol and significantly lower 24-h urinary norepinephrine to epinephrine ratios than non-suicidal patients (Ostroff et al. 1985). However, little is known about how and if ECT-associated alterations of (nor-) epinephrine and cortisol levels in blood affect the short- and long-term clinical outcome of patients suffering from MDD. Therefore, this exploratory study evaluates the acute and longitudinal effects of ECT on plasma (nor-) epinephrine and serum cortisol levels in relation to clinical outcome.

## Materials and methods

### Patients and treatment

Our prospective pilot study included 29 patients (9 male and 20 female) suffering from treatment-resistant MDD according to the ICD-10 (International Statistical Classification of Diseases and Related Health Problems 10th Revision) criteria. Patients who did not respond to two different, adequately applied antidepressants were considered as treatment-resistant. Depression severity was assessed before and after the first ECT as well as after the last ECT using the German versions of the Montgomery–Åsberg Depression Rating Scale (MADRS) and the revised version of the Beck Depression Inventory-II (BDI-II). Whereas a MADRS score below 10 was defined as remission (primary outcome), a reduction of > 50% was defined as response. To assess possible comorbid axis-II-disorders, the Structured Clinical Interview for Diagnostic II (SKID-II) and Statistical Manual of Mental Disorders, fourth Edition (DSM-IV) was performed.

Written informed consent was obtained from all patients before study inclusion. The Ethics Committee of Hannover Medical School (2842–2015) approved the study and it adhered to the Declaration of Helsinki (1964) and its later amendments.

All patients were treated as inpatients at the Department of Psychiatry, Social Psychiatry and Psychotherapy at Hannover Medical School (Germany). ECT administration in the facility was already described elsewhere (Moschny et al. 2020). In brief, right unilateral electrode placement and brief pulse ECT was used three times per week usually for 12 treatment sessions using a Thymatron IV device (Somatics, Lake Bluff, IL, USA). Anesthesia was obtained using methohexital or propofol, remifentanil and succinylcholine or mivacurium. Stimulation intensity was chosen depending on the patients’ age (age-based method). The energy was increased and the electrode placement was switched to bitemporal in case of non-responsiveness at the sixth session or if seizures were insufficient.

### (Nor-) epinephrine plasma levels and cortisol serum levels

Blood was withdrawn directly before (T1) and 15 min after (T2) the first and directly before the last ECT (T3) using 2 K EDTA-Gel and Serum S-Monovettes^®^ (Sarstedt AG & Co, Nümbrecht, Germany) as collection tubes. After temporal storage at 4 °C (up to 3 h) and room temperature (RT; 1 h, Serum S-Monovettes^®^ only), blood was centrifuged (2000×g, 10 min, RT: 2 K EDTA-Gel Monovettes^®^, 4 °C: Serum S-Monovettes^®^), aliquoted and kept at −80 °C until further use. The (nor-)/epinephrine levels were assessed using High Pressure Liquid Chromatography (HPLC). Cortisol was measured using an Electrochemiluminescence immunoassay (ECLIA) (Cobas 8000, module e801; Roche, Rotkreuz, Switzerland).

### Statistical analysis

Continuous variables are presented as mean ± standard deviation, categorical variables as numbers and percentages. Differences in baseline characteristics of the study population were assessed using *T* test or *χ*^*2*^ test with Yates correction. Repeatedly measured laboratory results were compared with the use of ANCOVA while considering age as a possible confounder. The assumption of equal variances was tested using the Levene’s test. After stratification *χ*^*2*^ test with Yates correction was performed to compare the changes of catecholamine levels between responders and non-responders. All hypothesis testing was two-tailed and *p* values of less than 0.05 were considered to indicate statistical significance. Bonferroni correction was applied for multiple testing. For data analysis, IBM SPSS Statistics for Windows, Version 26.0 (IBM Corp., Armonk, NY, USA) was used.

## Results

**Fig. 1:**
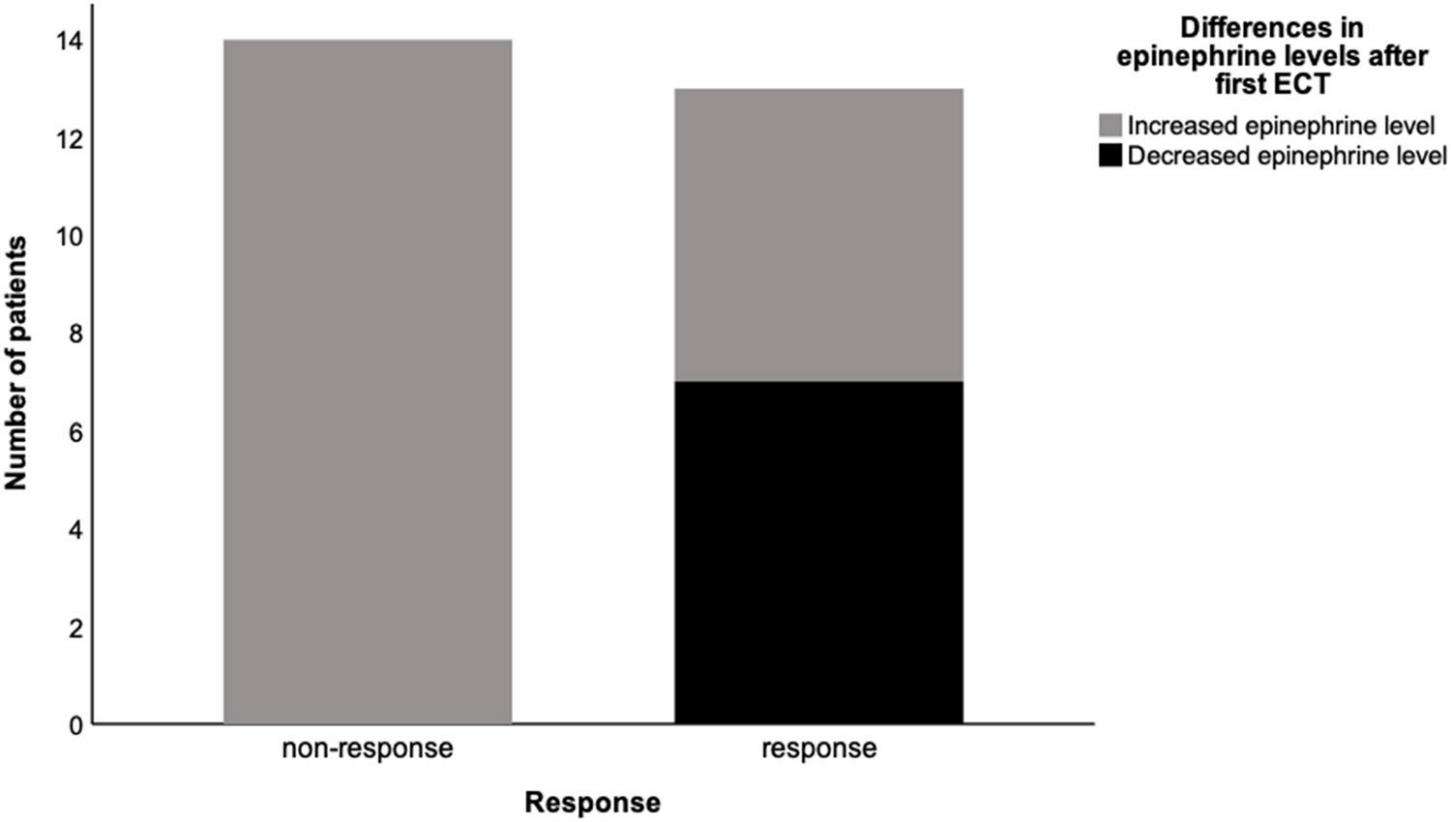
Difference in epinephrine levels before and shortly after first electroconvulsive therapy in relation to clinical outcome. Decrease and increase in epinephrine levels (ΔT1–T2) are displayed within non-responders versus responders

The patients’ baseline characteristics (*n* = 29, median age 54 years, 69% women) are presented in Table 1. At the end of the ECT series, 13 patients responded to ECT of which ten patients remitted. Responders and non-responders were comparable in their characteristics at baseline. Overall, a significant increase of epinephrine levels was observed shortly after the first ECT (Δ T1–T2, Bonferroni-corrected *p* value for comparison = 0.012). While in all non-responders an increase of epinephrine levels was measured (mean change ± standard deviation = 15.57 ± 11.30 ng/l), 7 of the 13 responders showed a decrease in their epinephrine levels (2.85 ± 17.88 ng/l; Fig. 1). After categorizing the patients according to the direction of the change (increase or decrease) a significant difference between responders and non-responders in the epinephrine levels was observed (*p* = 0.002). Responders in whom shortly after first ECT a decrease of epinephrine levels was measured had a significantly higher epinephrine level at T1 than responders with an increase of epinephrine levels (49.7 ± 22.3 versus 21.3 ± 8.7 ng/l, Bonferroni-corrected *p* = 0.014, Supplementary Table 1). Overall, before the last ECT (T3) epinephrine levels of all patients were comparable to baseline values (T1). However, after categorizing, responders with a decrease from T1 to T2 had tendentially lower epinephrine levels at T3 than at T1 (42.0 ± 21.3 and 49.7 ± 22.3 ng/l, respectively). Levels of cortisol performed similarly in responders and non-responders during the course of ECT. In both groups, the level of cortisol increased shortly after first ECT (T1 versus T2 Bonferroni-corrected *p* < 0.001) and decreased at T3 to levels comparable to baseline. Similarly, norepinephrine levels tended to increase at T2 but remained without significant changes throughout the ECT course. Overall, ECT had no significant effect on the long-term intraindividual deltas (difference from baseline to directly before the last ECT, ΔT1–T3) of epinephrine, norepinephrine, or cortisol (all three Bonferroni-corrected *p* = 1.000) and laboratory outcomes at T3 were, again, comparable between responders and non-responders (*p* = 0.848, *p* = 0.271 and *p* = 0.829 respectively, Supplementary Fig. 1).

**Table 1:** Patients’ characteristics at baseline

	Overall *n* = 29	Responder *n* = 13	Non-Responder *n* = 16	*P* value
Age, years	54 (± 15.6)	58 (± 11.7)	43 (± 19.8)	0.137
Women, *n* [%]	20 [69]	10 [67]	10 [71]	1.000
Body Mass Index, kg/m^2^	27 (± 6.5)	30.8 (± 5.4)	25 (± 6.2)	0.558
MADRS	33 (± 8.7)	39 (± 4.4)	34 (± 7.8)	0.314
BDI-II	36 (± 11.3)	46.0 (± 9.7)	38 (± 8.0)	0.199
Duration of current depressive episode, weeks	28 (± 20.1)	19.8 (± 13.0)	29.3 (± 24.7)	0.226
Age at initial diagnosis, years	32 (± 15.6)	30 (± 8.1)	37 (± 23.8)	0.938
History of suicide attempt, *n* [%]	9 [31]	3 [20]	6 [43]	0.245
Antidepressants, *n* [%]	17 [59]	10 [67]	7 [50]	0.462
Atypical antipsychotics, *n* [%]	13 [44.8]	8 [53]	5 [36]	0.462
Norepinephrine, ng/l	35 (± 19.8)	39.3 (± 35.7)	32.8 (± 10.8)	0.678
Epinephrine, ng/l	36 (± 18.8)	45.3 (± 33.1)	28.8 (± 11.4)	0.793
Cortisol, µg/dl	14 (± 6.2)	14.8 (± 1.6)	12.7 (± 5.5)	0.295

Patients’ characteristics presented as mean ± standard deviation (SD) or absolute quantity and percentual (*n* [%]); MADRS = Montgomery-Åsberg Depression Rating Scale, BDI-II = Beck Depression Inventory-II

## Discussion

The aim of the present study was to investigate the role of catecholamine plasma levels and cortisol serum levels in patients with treatment-resistant MDD receiving ECT and putting it into context with their clinical outcome. Our main finding was an acute decrease of the intraindividual epinephrine plasma level in patients responding to ECT. Norepinephrine plasma levels and cortisol serum levels, however, did not differ between ECT responders and non-responders. Regardless of clinical outcome, levels of cortisol in serum and norepinephrine in plasma were significantly elevated at T2 compared to T1 and seemed to normalize in the course of ECT.

It has previously been described that ECT has an effect on the autonomic nervous system and thus on norepinephrine release (Suzuki et al. 2019). Based on the diencephalon hypothesis ECT‐induced seizures must be sufficiently generalized in order to engage the diencephalic centers, which are associated with the regulation and modulation of physiological homeostasis, hormone release, appetitive behaviors, and diurnal rhythms. Furthermore, it is known that the diencephalon controls a variety of biological functions often implicated in depression through its hypothalamic releasing factors, involving the pituitary gland and autonomic nervous system (Suzuki et al. 2019). In a study from 1989 with a different ECT technique (bidirectional square waveform), an increase of cortisol, epinephrine and norepinephrine was shown in the first two minutes after the seizure (Mann et al. 1990). We were even able to find the increase of cortisol and norepinephrine 15 min after the seizure probably indicating the generalization of the seizure into the diencephalon in our patients.

Both the HPA axis and the noradrenergic network [with the latter starting from the Locus Coeruleus (LC)] are integral parts of the physiological stress response system and are thus implicated in cortisol release (Borodovitsyna et al. 2018). Chronic stress (a factor being repeatedly associated with depression) can activate the LC leading to norepinephrine release and subsequent activation of the HPA axis (Borodovitsyna et al. 2018). In the LC there are reciprocal stimulation mechanisms between corticotropin-releasing factor secreting cells and noradrenergic cells (Borodovitsyna et al. 2018). The prefrontal cortex (PFC) is a region found to show structural and functional changes in its communication with distal brain structures in patients suffering from depression (Hare and Duman 2020). The PFC has an inhibitory effect on the HPA axis and gets activated by slightly elevated norepinephrine levels. At high norepinephrine levels, however, the PFC is deactivated and the inhibitory effect disappears. PFC influences cortisol release via additional mechanisms, so that blocking the PFC can result in an uncontrolled cortisol stress response (Borodovitsyna et al. 2018). Cortisol, on the other hand, most likely inhibits norepinephrine release in the paraventricular nucleus of the hypothalamus, which is mainly supplied by the medulla and less by the LC (Borodovitsyna et al. 2018). In contrast, a study conducted with rats showed that cortisol increases norepinephrine levels in the LC, PFC, and striatum (Borodovitsyna et al. 2018), leading to the assumption of cortisol-induced norepinephrine release to be dependent on its region of origin. Additionally, an inhibitory role for norepinephrine in the HPA axis regulation in non-depressed persons is assumed. In addition, there seems to be a reciprocal effect for cortisol and noradrenergic activity (Goldstein and Kopin 2008). The regulation and feedback mechanisms could be disturbed in depressed patients. Sotsky and colleagues for example hypothesized that subjects with an increased noradrenergic responsivity during euthymic mood possibly deplete norepinephrine faster during a stressed state and therefore may lead to a disinhibition of the adrenocortical function (Sotsky et al. 1981). Additionally, dysregulation of the HPA axis induces both epinephrine and norepinephrine release via glucocorticoid-mediated stimulation in the adrenal medulla (Tank and Lee Wong 2015). The other involved system concerning physiological or environmental stress—the sympathetic nervous system (SNS)—is stimulated rapidly after stress with a subsequent release of epinephrine and norepinephrine from adrenal cells (Tank and Lee Wong 2015). Interestingly, epinephrine and norepinephrine responses are different depending on the type of stressor (Tank and Lee Wong 2015; Pacak et al. 1998). Overall, the SNS is considered to mediate short-term and immediate responses whereas the HPA axis is associated with the more sustained and long-term stress response (Tank and Lee Wong 2015). In our patients not responding to ECT catecholamine (meaning both norepinephrine and epinephrine) and cortisol levels increased after a single ECT, whereas in responders to ECT the epinephrine levels decreased. Our study was not designed to elucidate the causality of the observed differences between responders and non-responders, thus it can only be speculated that patients responding to ECT may have an altered stress response associated with an attenuated or time shifted release of epinephrine. Future studies could especially focus on the temporal dynamic of the HPA axis in ECT responders. Our patients did not suffer from hypercortisolism before ECT. Therefore, we were not able to confirm the normalization of the cortisol levels as suggested in multiple studies (Yuuki et al. 2005; Vukadin et al. 2011; Herman 2018; Yrondi et al. 2018).

Ostroff and colleagues described the epinephrine-norepinephrine-ratio (24-h urinary collection) in 99 male inpatients with mixed diagnoses to be significantly lower in patients who attempted suicide (Ostroff et al. 1985). We did not find a difference in those patients who had suicidal ideations in comparison to those without (data not shown). Ostroff and colleagues, however, only examined male patients and did not distinguish concerning response. Additionally, they did not differentiate between distinct diagnoses, and it thus remains unclear whether there was a difference in suicidal MDD patients (Ostroff et al. 1985).

Our findings encourage further investigation in the understanding of the catecholamine-metabolism in patients with treatment-resistant MDD receiving ECT. Especially suicidal ideations and suicidal attempts with respect to the catecholamine metabolism in MDD patients receiving ECT should be examined further.

Several limitations merit consideration. First, given the modest sample size the possibility of a type II error cannot be ruled out. Second, due to the only limited number of patients, this pilot study should be interpreted strictly exploratory. Third, in contrast to previous studies the catecholamine levels were obtained by measurements in the peripheral blood and not in 24-h urine (Ostroff et al. 1985). Therefore, and additionally due the inability of epinephrine to cross the blood brain barrier it remains elusive whether the central noradrenergic activity is adequately mirrored. Nevertheless, it is most likely that at least some CNS processes are reflected. Fourth, as possible drug discontinuation might have occurred a possible drug-induced impact on the response to ECT should be considered. However, the number of patients with antidepressants and atypical antipsychotics at baseline was comparable between the groups and it was common practice in the facility to keep the psychiatric medication stable throughout the course of ECT.

In conclusion all non-responders showed a higher epinephrine level at T2 as opposed to the patients who responded to treatment who had a significant decrease of epinephrine at T2 not only showing a short-term ECT effect on epinephrine levels but also suggesting that responders may have a subtype of a dysregulated HPA axis which is sensitive to ECT. Interestingly however, the observed differences only occurred in epinephrine (ΔT1–T2) but not in norepinephrine levels (ΔT1–T2).

## Supplementary Information

Below is the link to the electronic supplementary material.

Supplementary file1 (DOCX 5071 KB)

## Data Availability

The dataset used during the present study is available from the corresponding author on reasonable request.
