# Supplementary material for: Epinephrine levels decrease in responders after electroconvulsive therapy
Source: J Neural Transm (Vienna). 2021 Sep 23;128(12):1917–21. doi: 10.1007/s00702-021-02420-1 (PMC8571228; doi:10.1007/s00702-021-02420-1)

**Supplementary Material**

**Supplementary Table 1** **Comparison of baseline characteristics within responders**. Responders are stratified according to decrease and increase in epinephrine levels after the first electroconvulsive therapy (T1 and T2). Patients’ characteristics presented as mean ± standard deviation (SD) or absolute quantity and percentual (n [%]). MADRS = Montgomery-Åsberg Depression Rating Scale, BDI-II = Beck Depression Inventory-II.

| **Supplementary Table 1** | **Baseline Characteristics of the Patients** | | | | |
| --- | --- | --- | --- | --- | --- |
|  | Responders  n=13 | Decrease  n=7 | | Increase  n=6 | P-value |
| Age, years | 59 (± 11.6) | 61.7 (± 14.3) | 58.3 (± 10) | | 0.637 |
| Women, n [%] | 10 [77] | 5 [71] | 5 [83] | | 1.000 |
| Body Mass Index, kg/m² | 27 (± 6.1) | 15.7 (± 10.3) | 30.5 (± 7.1) | | 0.068 |
| MADRS | 34 (±10.1) | 34.7 (± 9.9) | 35.4 (± 5.2) | | 0.885 |
| BDI-II | 33 (± 11.3) | 36.7 (± 10.3) | 34.8 (± 11.9) | | 0.787 |
| Duration of current depressive episode, weeks | 21 (± 15.0) | 1.4 (± 1.2) | 0.8 (± 0.3) | | 0.046 |
| Age at initial diagnosis, years | 31 (± 13.8) | 32.8 (± 16.5) | 34.0 (± 12.5) | | 0.917 |
| History of suicide attempt, n [%] | 3 [23] | 3 [42] | 0 [0] | | 0.192 |
| Antidepressants, n [%] | 9 [69] | 5 [71] | 4 [66] | | 1.000 |
| Atypical antipsychotics, n [%] | 6 [46] | 4 [57] | 2 [33] | | 0.592 |
| Norepinephrine, ng/l | 36 (± 21.5) | 42.9 (± 28.3) | 29.7 (± 13.4) | | 0.321 |
| Epinephrine, ng/l | 37 (± 20.7) | 49.7 (± 22.3) | 21.3 (± 8.7) | | 0.014 |
| Cortisol, µg/dl | 14 (± 7.3) | 15.7 (± 10.3) | 12.6 (± 2.9) | | 0.486 |
|  |  |  |  | |  |

**Supplementary Figure 1. Comparison of epinephrine (A), norepinephrine (B) and cortisol (C) between responders and non-responders throughout the course** **of electroconvulsive therapy.** The estimated marginal means were calculated after adjustment for age as covariate using ANCOVA. The red line represents responders, while the blue line displays laboratory results of non-responders. Electroconvulsive therapy = ECT; timepoint 1 = directly prior to first ECT; timepoint 2 = 15 minutes after first ECT; timepoint 3 = shortly before last ECT.


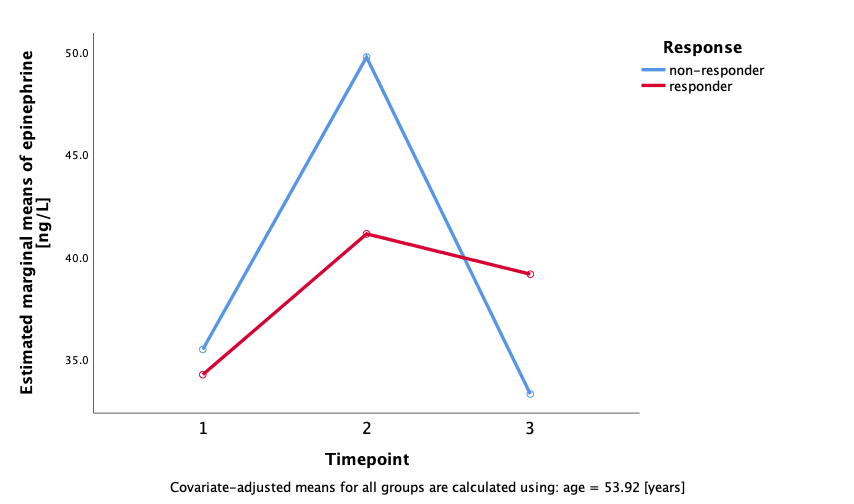


**C**

**B**

**A**


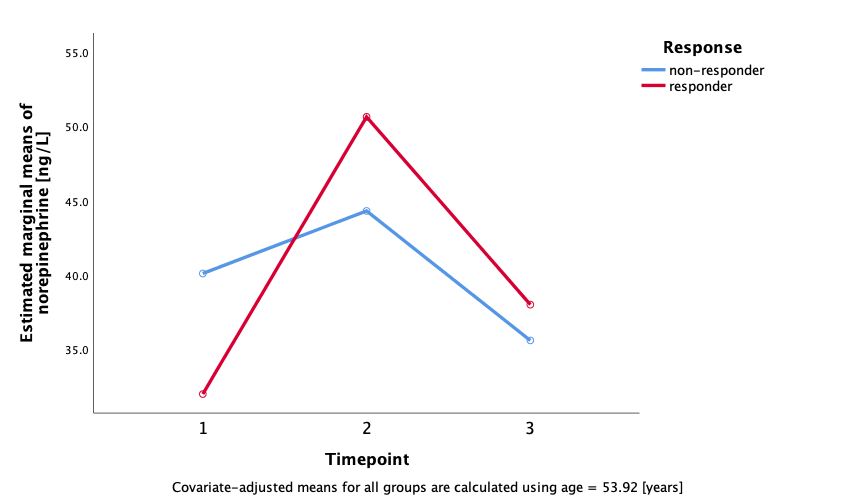


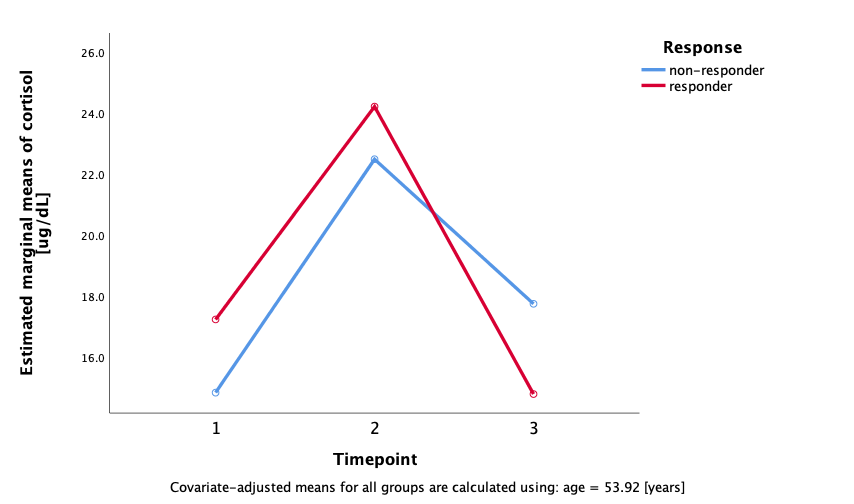

Supplement: Supplementary file 1 — Supplementary file1 (DOCX 5071 KB) [file 702_2021_2420_MOESM1_ESM.docx]
